# Young patients with congenital heart disease on psychotropic medications have higher recurrence of cardiac events than those without CHD

**DOI:** 10.3389/fcvm.2026.1771808

**Published:** 2026-03-18

**Authors:** Howaida Elmowafi, Sofia Edvinsson Sollander, Elin Risberg, Estelle Naumburg

**Affiliations:** Department of Clinical Sciences, Pediatrics, Umeå University, Umeå, Sweden

**Keywords:** cardiac events, congenital heart disease, pediatrics, psychotropic medications, recurrent cardiac events

## Abstract

**Background:**

Congenital heart disease (CHD) is a known risk factor for cardiac events, but its role in recurrent events among patients receiving psychotropic medications remains unclear. This study examines the impact of CHD on the risk of recurrent cardiac events in patients treated with psychotropic medications.

**Methods:**

This nationwide nested case–control study used Swedish register data from 2006 to 2018 and included individuals aged 5–30 years who were treated with psychotropic medications (attention-deficit hyperactivity disorder medications, antihistamines, antidepressants, anxiolytics, antipsychotics, and hypnotics) and experienced at least one cardiac event (e.g., cardiac arrest, arrhythmia, or syncope). Cases were individuals with recurrent cardiac events (≥2), while controls had a single event. The association between CHD and recurrent cardiac events was assessed by estimating adjusted odds ratios (AORs), controlling for sex, age, and psychotropic polypharmacy (≥2 medications).

**Results:**

A total of 23,504 individuals receiving psychotropic medical treatment experienced at least one cardiac event, of whom 2,409 (10.25%) had recurrent events. The median age was 20 years (IQR 17–24) for cases and 22 years (IQR 18–26) for controls. Most cases were female (70%). Both overall CHD and severe CHD were associated with a higher risk of recurrent cardiac events compared with patients without CHD (AOR 1.82; 95% CI 1.35–2.47 and AOR 1.77; 95% CI 1.29–2.42, respectively). Among patients with CHD, male sex, age 15–30 years, and absence of psychotropic polypharmacy were associated with a lower risk of recurrence.

**Conclusion:**

CHD is an independent risk factor for recurrent cardiac events among individuals treated with psychotropic medicines. Lower-risk factors included male sex, older age group, and absence of psychotropic polypharmacy.

## Introduction

Congenital heart disease (CHD) encompasses structural abnormalities of the heart or great vessels that arise during fetal development. CHD represents one of the most prevalent congenital malformations and affects about 1% of live births globally (1). Individuals with CHD often require surgical interventions and are at increased lifetime risk of cardiac events (2–5). Young patients with CHD have an increased risk of developing psychiatric and neurodevelopmental disorders, compared to their healthy peers (6, 7), and approximately 18 % of cardiac patients (including many with CHD) used psychotropic medications (8). Despite this risk profile, few safety aspects have been assessed for young patients with CHD undergoing psychiatric pharmacotherapy.

Nearly 1% of all children, adolescents, and young adults exposed to psychopharmacological treatment experience a cardiac event (9). Concerns regarding the cardiac safety of psychotropic medications have intensified in recent years (10–15). Some psychotropic medicines may induce severe cardiac events such as ventricular arrhythmias and sudden cardiac death (16–19). Psychotropic polypharmacy is a known risk factor for adverse drug events, including cardiac events (9, 20–24). Nevertheless, there remains a knowledge gap regarding whether specific psychotropic drug combinations, even those involving attention-deficit hyperactivity disorder (ADHD) medication, influence the risk of recurrent cardiac events in individuals exposed to CHD.

Patients with CHD are already predisposed to an elevated baseline risk of adverse cardiac events. We hypothesize that psychotropic medications may further contribute to this risk and that some factors may exert a protective effect, thereby attenuating the overall risk profile.

In this nested case–control study, we aimed to assess the association between recurrent cardiac events and CHD among individuals aged 5–30 years in Sweden who were treated with psychotropic medications. Furthermore, the study aimed to identify potential risk and protective factors associated with recurrence when ADHD medications are used in combination with other psychotropic drugs for individuals exposed to CHD.

## Materials and methods

### Study design

This is a nationwide, register-based nested case–control study, conducted in Sweden, utilizing data collected between 2006 and 2018. Patient data were obtained from the Swedish Prescribed Drug Register (SPDR) based on the Anatomical Therapeutic and Chemical (ATC) classification and from the National Patient Register (NPR) using the International Statistical Classification of Diseases and Related Health Problems, 10th version (ICD-10). Ethical approval was granted by the Swedish Ethical Review Board (Dnr 2019-04467 and Dnr 2020-05889).

### Study population

The study population comprised all individuals aged 5–30 years residing in Sweden who were exposed to psychotropic medications between 2006 and 2018 and had received a diagnosis of at least one predefined cardiac event after the exposure date (Supplementary Table S1).

Psychotropic medications included ADHD medications, antihistamines for systemic use, selective serotonin reuptake inhibitors (SSRIs), other antidepressants, anxiolytics, antipsychotics, and hypnotics.

Medication information was captured from the SPDR, while cardiac events were obtained from the NPR and included diagnosis of non-lethal cardiac arrest, arrhythmia, and syncope. Cardiac death was excluded, as this event can occur only once. The annual incidence of cardiac events among exposed individuals was based on the first event occurring each year throughout the study period.

### Selection of cases and controls

Cases were defined as individuals who experienced recurrent cardiac events (two or more events) during the study period, whereas controls were those who experienced only one cardiac event. A recurrent event could represent either a recurrence of the same event or the occurrence of a different type of event. Further details on the inclusion criteria for psychotropic medications, the included ATC codes, and cardiac events, included ICD codes, are provided in Supplementary Table S1.

### Exposure: congenital heart disease

Information on exposure, defined as the diagnosis of CHD, was obtained from the NPR and categorized into two subgroups: mild and severe, based on a well-known definition (25). Patients who initially received a diagnosis of mild CHD but were later diagnosed with severe CHD were classified in the severe CHD group. More information on the included ICD-10 codes and categorization is presented in Supplementary Table S1. Age was categorized into two groups: 5–14 and 15–30 years. Polypharmacy was defined as ≥1 day of concurrent use of two or more psychotropic medications.

### Statistical analysis

Population characteristics were reported as frequencies, percentages, or medians with interquartile ranges (IQRs), as appropriate. Statistical comparisons were performed using chi-square, Fisher's exact, or Mann–Whitney *U*-tests, depending on variable type and distribution. An assessment of the distribution of cardiac events across calendar-year quartiles was also conducted. In addition, the distribution of cardiac events among cases by event type was assessed. Associations between CHD (overall, mild, severe) and recurrent cardiac events were assessed using logistic regression, with adjustments for age, sex, and psychotropic polypharmacy. Results are presented as adjusted odds ratios (AORs) with 95% confidence intervals (CIs).

Additional subgroup analyses were performed to assess the association between CHD and recurrent cardiac events for specific psychotropic ATC classes, as well as for these classes in combinations with ADHD medication. Subgroups included each individual ATC category of psychotropic medication (antihistamines, SSRIs, other antidepressants, anxiolytics, antipsychotics, or hypnotics) and their combinations with ADHD medication.

Weighted risk scores were used to identify potential preventive and risk factors for cardiac event recurrence in patients with CHD. The scores were derived from a multivariable logistic regression model including the following predictors: CHD status (1 = no, 2 = yes), sex (1 = male, 2 = female), age groups (5–14 years, 15–30 years), and psychotropic polypharmacy (1 = no, 2 = yes). The reference group was switched into two separate analyses, and the resulting changes in estimates were evaluated. Regression coefficients from the model were used to compute individual risk scores. An additional analysis was carried out for patients whose CHD diagnosis was recorded before their first psychotropic prescription and first cardiac event. Finally, we conducted two analyses based on the recurrence of two or more events of the same type. One analysis evaluated recurrence of serious cardiac events (cardiac arrest, atrial arrhythmias, SVT, or VT), and the second evaluated recurrence of any arrhythmia (atrial arrhythmias, SVT, VT, or unspecified arrhythmia). These categorizations were used to increase statistical power.

The analyses were performed using Stata version 18 (College Station, TX: Stata Press) and SPSS.

## Results

**Figure 1:**
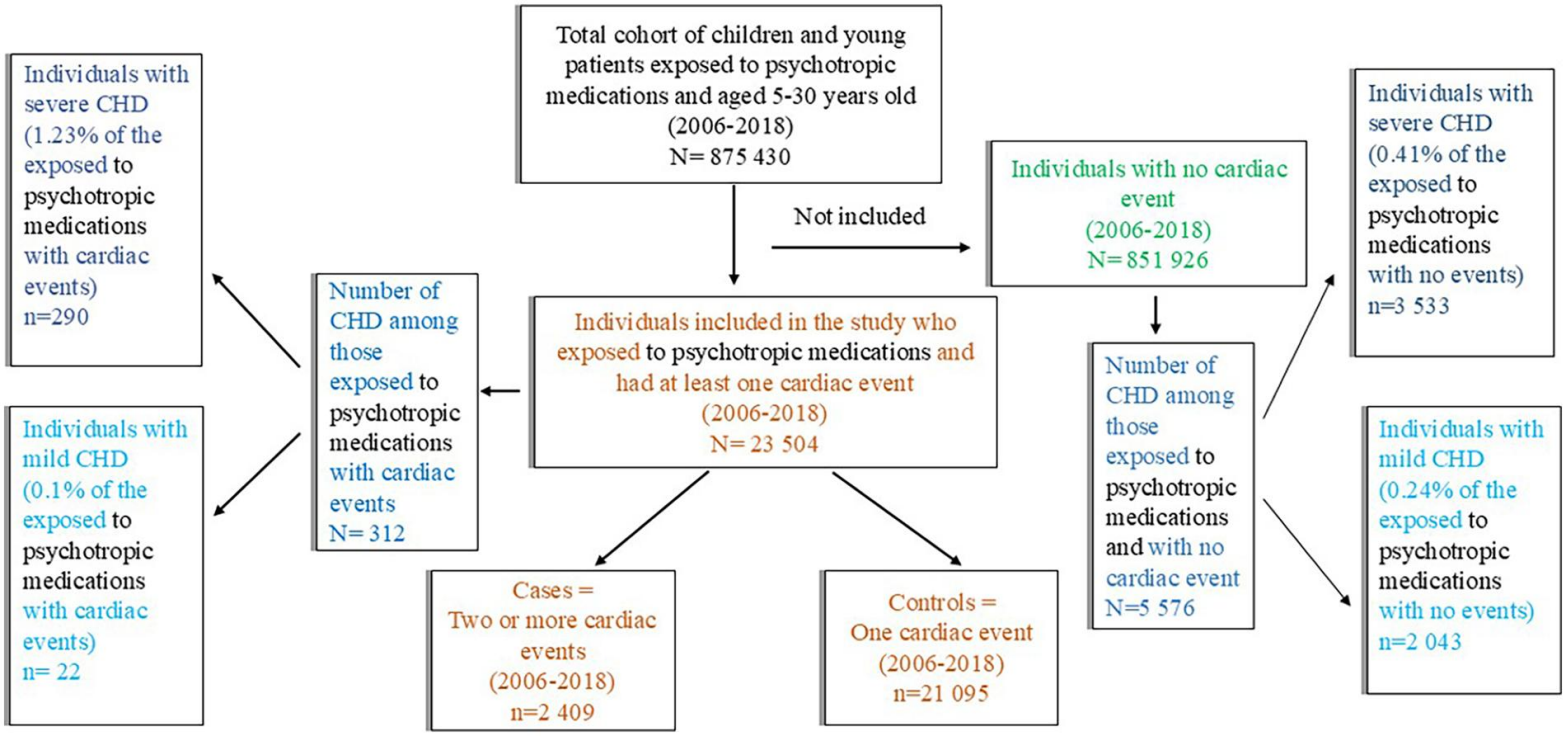
Distribution of the outcome (cardiac events) and exposure (congenital heart disease) among those exposed to psychotropic medications. Cardiac events included cardiac arrest, arrhythmias, and syncope. Psychotropic medication included attention deficit hyperactivity disorder medication, antihistamines for systemic use, antidepressants, anxiolytics, antipsychotics, hypnotics, and sedatives. Further details about ICD-10 and ATC codes are presented in the Supplementary Material.

Between 2006 and 2018, a total of 23,504 patients aged 5–30 years who were treated with psychotropic medications experienced at least one cardiac event. Of these, 2,409 (10.25%) were classified as cases (i.e., patients with recurrent cardiac events), and 21,095 (89.7%) were classified as controls (i.e., patients with a single event) (Figure 1). Among cases, cardiac events were more frequently distributed in the last calendar-year quartile (Supplementary Figure S1). Syncope was the most frequent cardiac event among cases, followed by unspecified arrhythmias (Supplementary Table S2).

Most included patients were female, with a higher proportion among cases (69.5%) than controls (62.4%). The median age (IQR) was 20 years (17–24) for cases and 22 years (18–26) for controls.

The prevalence of CHD exposure was higher among cases (2.2%) than among controls (1.2%) (*p* < 0.001). Most CHD cases were classified as severe types (92.9% of all CHD).

Psychotropic polypharmacy was also more common among cases (56.5%) than among controls (48.3%) (*p* < 0.001) (Table 1). Arrhythmias were more frequently observed among patients with a severe type of CHD (46.4%). Syncope was more commonly reported among patients without CHD (50.8%), followed by those with mild CHD. Ventricular tachycardia (VT) was identified in 15 patients with overall severe CHD (5.1%) but was not observed in those with mild CHD (Supplementary Table S3).

**Table 1:** Characteristics of cases and controls.

Variables	Cases *N* = 2,409	Controls *N* = 21,095	*P*-values
Sex			<0.001^a^
Male *n* (%)	736 (30.55)	7, 938 (37.63)	
Female *n* (%)	1, 673 (69.45)	13, 157 (62.37)	
Age, median (IQR), years	20 (17–24)	22 (18–26)	<0.001^b^
Age groups			<0.001^a^
5–14 *n* (%)	326 (13.53)	2, 334 (11.06)	
15–30 *n* (%)	2, 083 (86.47)	18, 761 (88.94)	
Congenital heart disease (CHD)			<0.001^a^
No, *n* (%)	2, 357 (97.84)	20, 835 (98.77)	
Yes, *n* (%)	52 (2.16)	260 (1.23)	
CHD by type			<0.001^c^
CHD, *n* (mild %)	5 (0.21)	17 (0.08)	
CHD, *n* (severe %)	47 (1.95)	243 (1.15)	
Polypharmacy			<0.001^a^
Polypharmacy, *n* (no %)	1, 049 (43.55)	10, 897 (51.66)	
Polypharmacy, *n* (yes %)	1, 360 (56.45)	10, 198 (48.34)	

IQR, interquartile range; CHD, congenital heart disease, ICD Q20–Q26. Mild, Q21.4, Q24.6, Q25.0, Q25.5; Severe, Q20–Q26 and Q27.8, excluding ICD codes for “mild.” Polypharmacy refers to the exposure to two or more psychotropic drugs.

Cases were patients aged 5–30 years who had been exposed to psychotropic medications and developed more than one cardiac event (recurrent events) during the study period 2006–2018.

Controls were patients aged 5–30 years who had been exposed to psychotropic medications and developed one cardiac event during the same study period.

^a^ *P*-value calculated using the chi^2^ test.

^b^ *P*-value calculated using Mann–Whitney *U*-test.

^c^ *P*-value calculated using Fisher's exact test.

An association was found between CHD and recurrent cardiac events (AOR 1.82; 95% CI: 1.35–2.47). Severe types of CHD were associated with an increased risk of recurrent cardiac events (AOR 1.77; 95% CI: 1.29–2.42), while there was no association between mild CHD and recurrent cardiac events (AOR 2.61; 95% CI: 0.95–7.11) (Table 2).

**Table 2:** Association between congenital heart disease and recurrent cardiac events.

Variables	Crude odds ratio (95% CI)	Adjusted for age (95% CI)	Adjusted for sex (95% CI)	Fully adjusted odds ratio (95% CI)
CHD-all				
No	1 (Ref)	1 (Ref)	1 (Ref)	1 (Ref)
Yes	1.77 (1.31–2.39)	1.73 (1.28–2.34)	1.78 (1.32–2.41)	1.82 (1.35–2.47)
Association by severity of CHD				
No	1 (Ref)	1 (Ref)	1 (Ref)	1 (Ref)
Mild	2.60 (0.96–7.05)	2.46 (0.90–6.68)	2.47 (0.89–6.62)	2.61 (0.95–7.11)
Severe	1.71 (1.25–2.34)	1.68 (1.22–2.30)	1.73 (1.26–2.37)	1.77 (1.29–2.42)

CHD, congenital heart disease, ICD Q20–Q26; No, no CHD; Yes, yes CHD; Mild, Q21.4, Q24.6, Q25.0, Q25.5; Severe, Q20–Q26 and Q27.8, excluding ICD codes for “mild.” Polypharmacy refers to the exposure to two or more psychotropic medications. CI, confidence interval. The fully adjusted odds ratios were obtained after adjustment for age groups (5–14, 15–30), sex, and psychotropic polypharmacy. Ref, reference group. Associations with mild CHD were not significant due to the few patients with mild CHD (i.e., the analyses are exploratory and likely underpowered).

**Figure 2:**
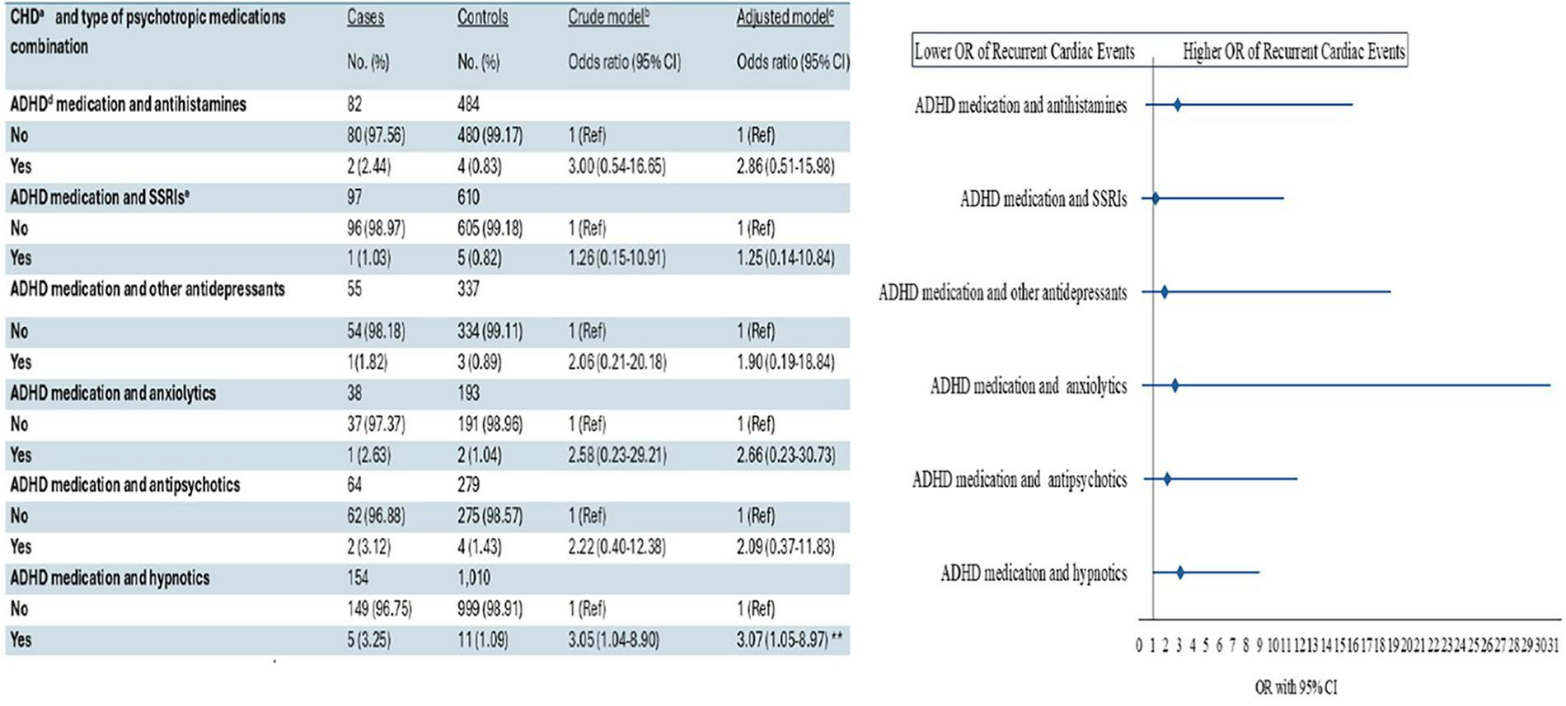
Association between congenital heart disease and recurrent cardiac events, stratified by type of psychotropic medication combination.

A possible association between recurrent cardiac events and CHD was observed among patients receiving ADHD medication in combination with hypnotics (AOR 3.07; 95% CI: 1.05–8.97). No other associations were found between overall CHD and recurrent cardiac events across the stratified psychotropic drug classes or in combinations of other psychotropics with ADHD medication (Figure 2).

**Figure 3:**
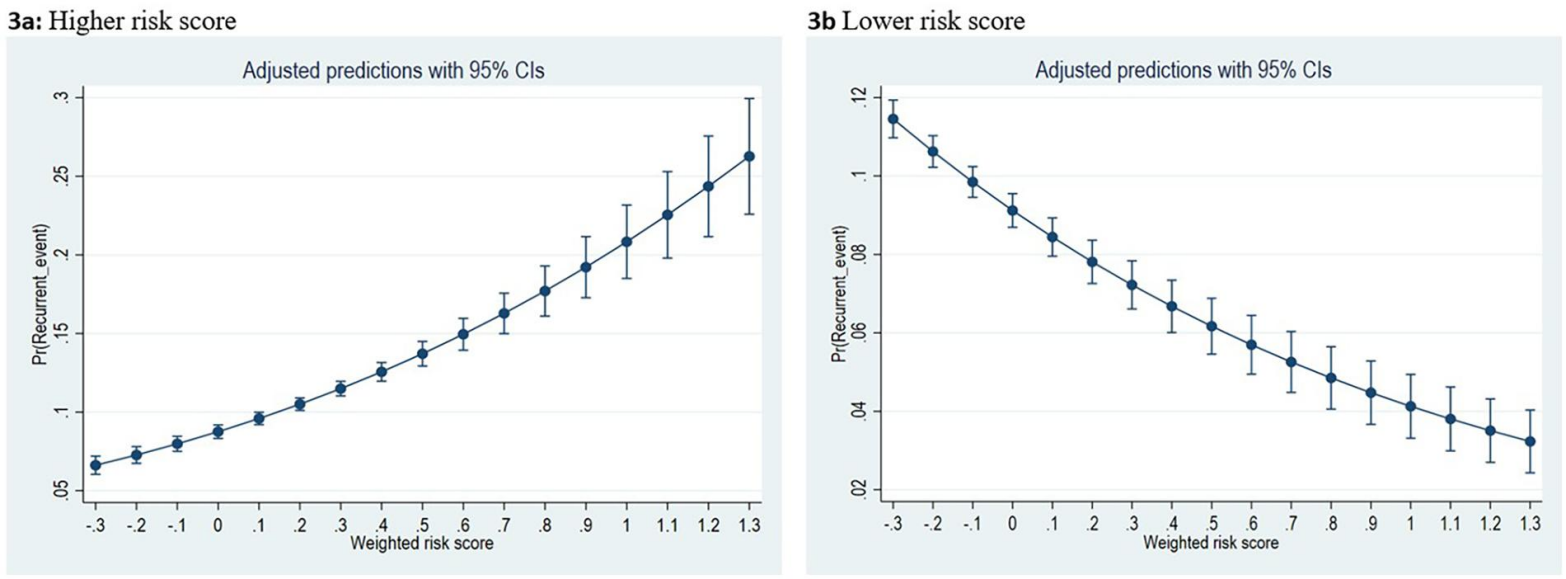
Risk prediction of recurrent cardiac events in congenital heart disease (CHD) patients exposed to psychotropic medications using a weighted risk score. The weighted risk was calculated using regression coefficients derived from a multivariable logistic regression model. Included predicators were CHD (1 = no, 2 = yes), sex (1 = male, 2 = female), age groups (5-14 and 15-30 years), and psychotropic polypharmacy (1 = no, 2 = yes). In **(a)**, we use the first category of the included variable as reference group. Female sex, young age group and use of psychotropic pharmacy contributed to higher risk score in CHD patients. In **(b)**, we used the second category of the variable as the reference group to evaluate changes in the risk of cardiac event reference in CHD patients. Male sex, older age group and using a single psychotropic medication decreased the risk of recurrent cardiac events in patient with CHD.

Higher weighted risk scores were significantly associated with recurrence of cardiac events in patients with CHD. Among female patients of younger age taking multiple psychotropic medications, each 1-unit increase in the cumulative weighted risk score was associated with a 2.74-fold increase in the odds of recurrence of cardiac events (AOR 2.74; 95% CI: 2.32–3.25). The predicted probability of recurrence increased steadily as risk scores increased. Conversely, when using male sex, older age groups, or patients taking a single psychotropic medication as the reference categories, higher risk scores were associated with lower odds of recurrence of cardiac events (AOR 0.43; 95% CI: 0.36–0.51) in patients with CHD. That is, each 1-unit increase in the risk score corresponded to a nearly 57% decrease in the odds of a recurrent event. Based on these factors, the predicted probability of recurrence declined among these groups (Figures 3a,b). The results were similar when the analysis was restricted to patients who received a CHD diagnosis before the exposure (AOR 1.88; 95% CI: 1.13–3.14). A significant association was observed for serious cardiac events (AOR 4.45; 95% CI: 2.97–6.69) and for overall arrhythmias among those who experienced events of the same type (Supplementary Table S4).

## Discussion

In this nationwide nested case–control study, we found that CHD is associated with an 82% increased risk of recurrent cardiac events in children, adolescents, and young adults treated with psychotropic medications, mainly attributable to severe CHD. ADHD medication in combination with hypnotics was linked to a modest elevation in risk. Factor associated with a lower risk of recurrence of cardiac events were male sex, age group 15–30, and absence of psychotropic polypharmacy. These findings provide important epidemiological evidence to inform risk stratification and monitoring priorities for psychotropic use in young patients with CHD and underscore the need for close integration of cardiology and psychiatric care when psychotropic medications are prescribed in this population.

Patients with CHD are at a significantly higher risk of arrhythmia than the general population, especially among those with complex CHD, contributing to markedly increased health care utilization and mortality (2–5, 26). Furthermore, CHD frequently coexists with psychiatric and neurodevelopmental conditions, including ADHD, particularly in patients with severe forms of CHD. Overall, more than 35% of pediatric patients with CHD are affected by a psychiatric disorder. The coincidence of CHD and mental health disorders is associated with an increased risk of complications, adversely affecting both cardiovascular and psychiatric outcomes (6, 27, 28). Medications used to manage these psychiatric conditions may further increase cardiovascular risk in this vulnerable population of patients with CHD (29). These reports not only support and help explain our findings but also align with previous research showing that patients with CHD are more vulnerable to adverse drug reactions, particularly cardiovascular events, compared with individuals without CHD (30, 31). Therefore, we argue for careful baseline assessment and close monitoring (e.g., ECG) for patients with CHD using psychotropic medicines, particularly those with severe types of CHD.

The association between mild CHD and recurrent cardiac events among patients with psychotropic medications did not reach statistical significance in our study, which is inconsistent with others (32–35). However, this may be explained by the small number of patients with mild CHD in our study, differences in inclusion criteria, or the older age groups included in previous research. This may also reflect differences in outcomes, as most previous studies evaluated the risk of the first cardiac event, whereas our study assessed the risk of recurrence in patients with CHD, who are known to have a high baseline risk of arrhythmia.

Our findings should be interpreted with caution and cannot be considered as evidence of the safety of psychotropic medication in this population. Further studies with larger sample sizes might be able to estimate the effect among patients with less severe forms of CHD.

The absence of association between CHD and recurrent cardiac events when stratifying by psychotropic medication class doed not align with the well-documented and varied cardiac risks associated with several psychotropic drug classes, including SSRIs, hypnotics, and antipsychotics, among individuals without CHD (11, 17–19, 29, 36). This may be due to clinicians opting to avoid certain types of psychotropic medications in patients with CHD (37).

We found no significant associations between CHD and the risk of recurrent cardiac events in subgroup analyses of ADHD medication in combination with other psychotropic medicines, except for the combination of ADHD medication and hypnotics. Although potential pharmacokinetic and pharmacodynamic interactions between ADHD medication and other psychotropic agents could plausibly influence cardiac risk (20–24), the lack of statistical significance in our nationally based study may reflect the relatively limited sample sizes within these subgroups. Therefore, these findings should be interpreted cautiously and considered exploratory. Nonetheless, they highlight the potential value of individualized risk stratification and multidisciplinary monitoring when prescribing such combinations.

ADHD medication use was common in both groups—patients with CHD and patients without CHD—but the CHD group had a slightly lower proportion (19% vs. 21%). Current clinical guidelines recommend that children with known cardiac symptoms or risk factors should avoid pharmacological intervention with stimulant ADHD treatment or receive reduced dosages (37, 38). Thus, the increased association between severe CHD and recurrent events observed in our study may potentially be underestimated, as we believe that many young patients with cardiac symptoms or disease may not receive medical treatment for ADHD. However, risk stratifications and preventive measures regarding psychotropic medicines, or their combinations, in this group of patients remain limited. We hope that our study will contribute to this knowledge gap.

Our findings indicate that female patients, younger individuals, and those with CHD receiving psychotropic polypharmacy are particularly vulnerable to recurrent cardiac events. These findings are consistent with previous research identifying female sex and polypharmacy as risk factors for overall drug-induced adverse events (31, 39, 40). However, we found that male sex, older age group (15–30 years old), and absence of psychotropic polypharmacy were associated with a lower risk score. While biological susceptibility may partly explain this finding, gender-related differences in healthcare-seeking behavior and treatment could also have influenced the observed effect (31, 41). Identifying individual risk factors is valuable for targeted prevention, individualized risk–benefit assessment, and the implementation of multidisciplinary care. Clinicians should exercise heightened vigilance and avoid unnecessary psychotropic polypharmacy.

### Strengths and limitations

This nationwide study used real-world data from an existing cohort of patients exposed to psychotropic medications. By utilizing mandatory health care registration in Sweden, the study benefits from high coverage and completeness, thereby enhancing external validity and limiting the risk of selection bias.

We included the first cardiac event for each patient during each calendar year, from 1January 2006 to 31 December 2018. Analysis of cardiac events across calendar-year quartiles showed a consistent distribution for controls (Supplementary Figure S1), suggesting that restricting the analysis to the first annual event did not introduce selection bias related to time of year and ensured that events occurring later in the year were not systematically excluded.

However, we observed a slight increase in cardiac event registrations during the fourth quarter (Q4) of the year among cases (Supplementary Figure S1). The cause of this increase is unclear. One possible explanation may relate to treatment protocols during the study period, suggesting a temporary discontinuation of psychotropic medications, especially ADHD medications, during summer holidays, followed by resumption later in the year. However, this hypothesis is not supported by a similar pattern among controls, and therefore, we believe that this did not introduce a bias into our results. Confounding by indication and/or detection bias may have taken place due to differences in treatment choices and the intensity of follow-up over time for patients with CHD.

The study excluded fatal cardiac events because cardiac death is a non-recurrent outcome; however, this exclusion may introduce survival bias if patients with CHD are more likely to die at their initial cardiac event and are therefore omitted from the recurrent-event analysis. In the source population, there were 13 deaths among exposed individuals (9), but only four deaths occurred among those who experienced a cardiac event. Therefore, we believe that any resulting bias is limited.

A key limitation of our study is the lack of adjustment for socioeconomic and lifestyle factors, which may have introduced residual confounding. Additionally, the small number of individuals in the younger age group (13.5% of cases) and those included in the medication combination subgroup analyses limited our ability to draw definitive conclusions for these groups. These analyses, of course, were exploratory and, despite the nationwide approach and large sample size, remained underpowered. A larger sample size would improve the precision of these estimates and allow for more robust subgroup analyses.

## Conclusions

This nationwide nested case–control study identified a significant association between severe CHD and the risk of recurrent cardiac events among individuals exposed to psychotropic medications. The combination of ADHD medication and hypnotics may be associated with a modestly elevated risk. Female sex, younger age, and psychotropic polypharmacy may represent potential risk factors in the presence of CHD. Factors associated with a lower likelihood of recurrent cardiac events were male sex, older age, and exposure to only one type of psychotropic medication. These findings emphasize the importance of personalized risk–benefit evaluation in patients with CHD receiving psychotropic medications.

## Data Availability

The data analyzed in this study are subject to the following licenses/restrictions: the data that support the findings of this study are available in Swedish from Socialstyrelsen, the Swedish National Board of Health and Welfare, but restrictions apply. The data were used under license for the current study and are not publicly available. Requests to access these datasets should be directed to https://bestalladata.socialstyrelsen.se/.
