# Supplementary material for: Young patients with congenital heart disease on psychotropic medications have higher recurrence of cardiac events than those without CHD
Source: Front Cardiovasc Med. 2026 Mar 18;13:1771808. doi: 10.3389/fcvm.2026.1771808 (PMC13038961; doi:10.3389/fcvm.2026.1771808)
Supplement: Supplementary file 1 [file Datasheet1.docx]

Table S1. ICD-10 codes included, for the outcome and exposure-congenital heart disease as well as ATC codes for psychotropic medications.

| **Outcome-cardiac event** | ICD-10 codes |
| --- | --- |
| Cardiac arrest unspecified | I46.9 |
| Reentry ventricular tachycardia | I47.0 |
| Paroxysmal supraventricular tachycardia | I47.1 |
| Ventricular tachycardia | I47.2 |
| Ventricular tachycardia, torsades de pointes | I47.2C |
| Ventricular fibrillation and ventricular flutter | I49.0 |
| Premature depolarization of atrial ventricular flutter non-specified | I49.1 |
| Premature depolarization of the ventricle | I49.3 |
| Sick sinus syndrome | I49.5 |
| Brady tachy syndrome | I49.5C |
| Other specified cardiac arrhythmias | I49.8 |
| Cardiac replacement rhythm | I49.8C |
| Cardiac arrhythmia non-specified | I49.9 |
| Non-specified | R00 |
| Fainting and collapse | R55.9 |
| **Risk factor- congenital heart disease (CHD)** | |
| **CHD severe** |  |
| **Q20.0-Q20.9 (excluding Q20.7).** |  |
| Truncus communis | Q20.0 |
| Double outflow from R ventricle | Q20.1 |
| Double outflow from L ventricle | Q20.2 |
| Discordant ventriculoarterial connection | Q20.3 |
| Double inflow to ventricle | Q20.4 |
| Discordant atrioventricular connection | Q20.5 |
| Atrial isomerism | Q20.6 |
| Other specified congenital malformation of heart ventricles and connections | Q20.8 |
| Congenital malformation of heart ventricles and connections, unspecified | Q20.9 |
| **Q21.2-Q21.3** |  |
| Atrioventricular septum defect | Q21.2 |
| Tetralogy of Fallot | Q21.3 |
| Q22.0-Q22.9 (excluding Q22.2, Q22.3, Q22.7) |  |
| Pulmonary valve atresia | Q22.0 |
| Congenital stenosis of pulmonary valve | Q22.1 |
| Congenital tricuspid valve stenosis | Q22.4 |
| Ebsteins anomaly | Q22.5 |
| Hypoplastic right heart syndrome | Q22.6 |
| Other congenital malformation of the tricuspid valve | Q22.8 |
| Congenital malformation of tricuspid valve, unspecified | Q22.9 |
| **Q23.0-Q23.9 (excluding Q23.5, Q23.6, Q23.7)** |  |
| Congenital aortic valve stenosis | Q23.0 |
| Congenital aortic valve insufficiency / bicuspid aortic valve | Q23.1 |
| Congenital mitral valve stenosis | Q23.2 |
| Congenital mitral valve insufficiency | Q23.3 |
| Hypoplastic left ventricle syndrome | Q23.4 |
| Other specified congenital malformations of the aortic valve and mitral valve | Q23.8 |
| Congenital malformation of aortic valve and mitral valve, unspecified | Q23.9 |
| **Q24.2-Q24.9 (excluding Q24.3, Q24.7)** |  |
| Cor triatriatum | Q24.2 |
| Congenital subaortic stenosis | Q24.4 |
| Coronary arterial malformation | Q24.5 |
| Other specified congenital heart malformations | Q24.8 |
| Congenital heart malformation, unspecified | Q24.9 |
| **Q25.1-Q25.9** |  |
| Coarctation of aorta | Q25.1 |
| Aortic atresia | Q25.2 |
| Aortic stenosis | Q25.3 |
| Other congenital aortic malformations | Q25.4 |
| Stenosis of pulmonary artery | Q25.6 |
| Other congenital malformations of the pulmonary artery | Q25.7 |
| Other specified congenital malformations of the great arteries | Q25.8 |
| Congenital malformation of the great arteries, unspecified | Q25.9 |
| **Q26.0-Q26.9** |  |
| Congenital stenosis of vena cava | Q26.0 |
| Remaining upper left vena cava sup | Q26.1 |
| Total anomalous pulmonary venous outflow | Q26.2 |
| Partially anomalous pulmonary venous outflow | Q26.3 |
| Anomalous pulmonary venous connection, unspecified | Q26.4 |
| Anomaly of the vena porta connection | Q26.5 |
| Fistula between vena porta and hepatic artery | Q26.6 |
| Other specified congenital malformations of the greater veins | Q26.8 |
| Congenital malformation of the greater veins, unspecified | Q26.9 |
| Other specified congenital malformations of the peripheral vascular system | Q27.8 |
| **CHD mild:** All other CHD codes excluding those for severe types. | |
| Aortopulmonary septum defect | Q21.4 |
| Congenital heart block | Q24.6 |
| Patent ductus arteriosus | Q25.0 |
| Open ductus arteriosus | Q25.5 |
| ICD-10: The International Classification of Disease and Related Health Problems- 10^th^ edition. | |

| Continue Table S1 | |
| --- | --- |
| **Psychotropic medications** | ATC codes |
| ADHD drugs ^a^ |  |
| Methylphenidate | N06BA04 |
| Dexamphetamine | N06BA02 |
| Lis dexamphetamine | N06BA12 |
| Atomoxetine | N06BA09 |
| Guanfacine | C02AC02 |
| Antihistamines |  |
| Alimemazine | R06AD01 |
| Promethazine | R06AD02 |
| Promethazine, combination | R06AD52 |
| Anti-depressants SSRIs ^b^ ­ |  |
| Fluoxetine | N06AB03 |
| Citalopram | N06AB04 |
| Paroxetine | N06AB05 |
| Sertraline | N06AB06 |
| Fluvoxamine | N06AB08 |
| Escitalopram | N06AB10 |
| Other antidepressants |  |
| Clomipramine | N06AA04 |
| Amitriptyline | N06AA09 |
| Nortriptyline | N06AA10 |
| Moclobemide | N06AG02 |
| Mianserin | N06AX03 |
| Mirtazapine | N06AX11 |
| Bupropion | N06AX12 |
| Venlafaxine | N06AX16 |
| Reboxetine | N06AX18 |
| Duloxetine | N06AX21 |
| Agomelatine | N06AX22 |
| Vortioxetine | N06AX26 |
| Esketamine | N06AX27 |
| Antipsychotics |  |
| Levomepromazine | N05AA02 |
| Perphenazine | N05AB03 |
| Haloperidol | N05AD01 |
| Melperone | N05AD03 |
| Droperidol | N05AD08 |
| Sertindole | N05AE03 |
| Ziprasidone | N05AE04 |
| Lurasidone | N05AE05 |
| Flupentixol | N05AF01 |
| Chlorprothixene | N05AF03 |
| Zuclopenthixol | N05AF05 |
| Clozapine | N05AH02 |
| Olanzapine | N05AH03 |
| Quetiapine | N05AH04 |
| Lithium | N05AN01 |
| Risperidone | N05AX08 |
| Aripiprazole | N05AX12 |
| Paliperidone | N05AX13 |
| Cariprazine | N05AX15 |
| Anxiolytics (benzodiazepines) |  |
| Diazepam | N05BA01 |
| Oxazepam | N05BA04 |
| Lorazepam | N05BA06 |
| Alprazolam | N05BA12 |
| Others including hypnotics and sedatives |  |
| Hydroxyzine | N05BB01 |
| Buspirone | N05BE01 |
| Zopiclone | N05CF01 |
| Zolpidem | N05CF02 |
| Melatonin | N05CH01 |
| Clomethiazole | N05CM02 |
| Propiomazine | N05CM06 |
| ^a^ ADHD: Attention deficit hyperactivity disorder ATC: The Anatomical Therapeutic and Chemical Classification  ^b^ SSRIs ­: selective serotonin reuptake inhibitors. | |

Figure S1. Pattern of cardiac events among the exposed individuals- cases and controls

| 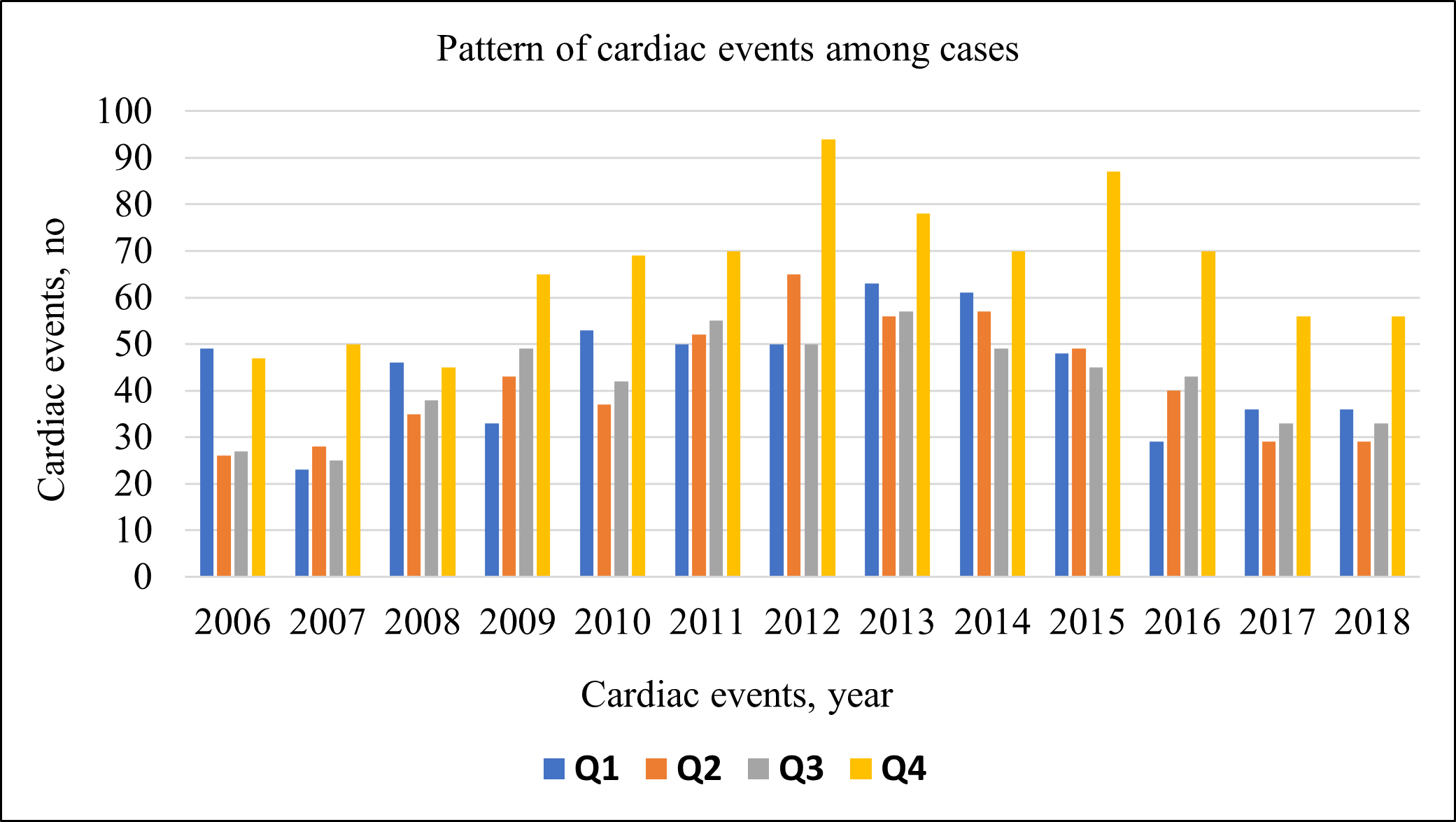 |
| --- |
| 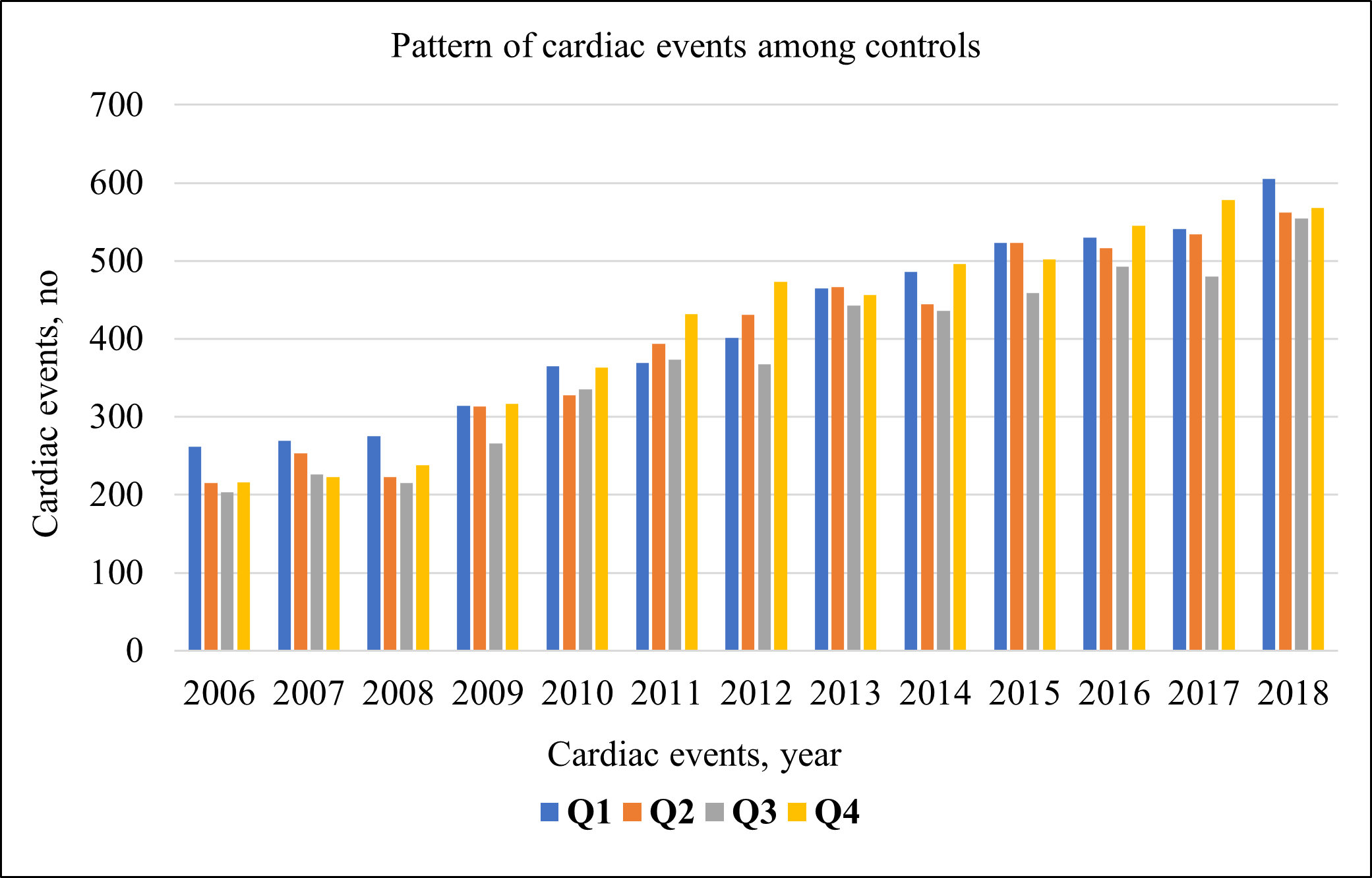 |
| Cases= Individuals aged 5-30 years who were exposed to psychotropic medications and had more than one cardiac event during 2006-2018.  Q1, Q2, Q3, Q4 are the quartiles of the year.  Controls= Individuals aged 5-30 years who were exposed to psychotropic medications and had only one cardiac event during the same study period. |

| Table S2. Type of recurrent cardiac events among cases | | |
| --- | --- | --- |
| Event type | Events  N= 5 655 |  |
| Cardiac arrest n (%) | 18 (0.32%) |  |
| SVT n (%) | 530 (9.37%) |  |
| VT n (%) | 298 (5.27%) |  |
| Atrial arrhythmias n (%) | 29 (0.51) |  |
| Non-specified arrhythmias n (%) | 2 193 (38.78%) |  |
| Syncope n (%) | 2 587 (% 45.75) |  |
| Cardiac arrest = resuscitated cardiac arrest. SVT = super ventricular tachycardia.  VT = ventricular tachycardia.  % was calculated as column percentage from number of events  Number of unique individuals with recurrent events = 2 409, but the number of events was 5 655 | | |

| Table S3. The type of first cardiac event, by congenital heart disease severity. | | | |
| --- | --- | --- | --- |
| Event type | No CHD | Mild CHD | Severe CHD |
| Cardiac arrest n (%) | 264 (1.13%) | 2 (9.09%) | 11 (3.75%) |
| SVT n (%) | 671 (2.88%) | 4 (18.18%) | 21 (7.17%) |
| VT n (%) | 505 (2.17%) | 0 (0%) | 15 (5.12%) |
| Atrial arrhythmias n (%) | 39 (0.17%) | 2 (9.09%) | 8 (2.73%) |
| Non-specified arrhythmias n (%) | 9 978 (42.89%) | 6 (27.27%) | 136 (46.42%) |
| Syncope n (%) | 11 807 (50.75%) | 8 (36.36%) | 102 (34.81%) |
| CHD = congenital heart disease, ICD Q20-Q26. Mild; Q21.4, Q24.6, Q25.0, Q25.5. Severe; Q20-Q26 and Q27.8, minus ICD-code for “mild”. Cardiac arrest includes resuscitated cardiac arrest. SVT = super ventricular tachycardia. VT = ventricular tachycardia.  % was calculated as row percentage. | | | |

| Table S4. Risk of recurrent cardiac events associated with congenital heart disease (CHD) by same type of event (recurrent event of same type) | | |
| --- | --- | --- |
|  | Crude odds ratios,  95% CI | Adjusted odds ratios, 95% CI |
| Serious event |  |  |
| No | 1 (Ref) | 1 (Ref) |
| Yes | 4.53 (3.02-6.79) | 4.45 (2.97-6.69) |
| Any type of arrhythmias | | |
| No | 1 (Ref) | 1 (Ref) |
| yes | 2.19 (1.57-3.07) | 2. 26 (1.61-3.17) |
| Serious event = Cardiac arrest, SVT (super ventricular tachycardia), VT (ventricular tachycardia and atrial arrhythmias.  = atrial arrythmia, SVT, VT and non-specified cardiac arrhythmia.  Any type of arrhythmia = atrial, SVT, VT and unspecified arrhythmia  No = no CHD Yes = yes CHD | | |
